# Loose Anagen Hair Syndrome in a Saudi Girl

**DOI:** 10.5826/dpc.1104a071

**Published:** 2021-10-01

**Authors:** Mahdi Al Dhafiri, Muneerah Alhumaidy

**Affiliations:** 1 Dermatology Department, College of Medicine, King Faisal University, AlAhsa, Saudi Arabia; 2 College of Medicine, King Faisal University, AlAhsa, Saudi Arabia

**Keywords:** loose anagen hair syndrome, black, hair, Saudi, children

## Introduction

Loose anagen hair syndrome (LAHS) is an uncommon, self-limited condition mainly affecting young children with a fair phototype and light hair. It has a female predominance, the average age of onset is three years old. It is characterized by slowly growing hair and sparse scalp hair that is easily and painlessly pulled out. LAHS is related to the premature keratinization of the inner root sheath [1]. Herein, we present a case of a black-haired young girl of Saudi origins with LAHS.

## Case Presentation

**Figure 1:**
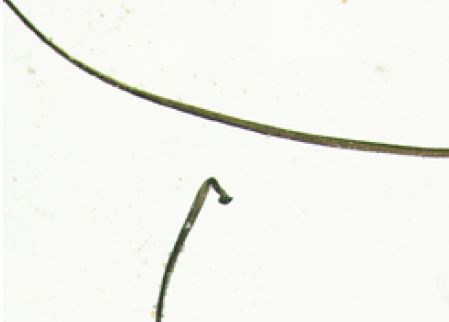
Light microscopy examination showing the hair’s characteristic feature in loose anagen hair syndrome with misshapen bulbs and ruffled cuticles.

**Figure 2:**
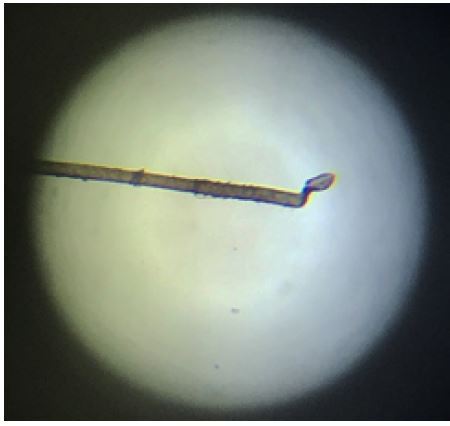
Light microscopy examination showing the hair’s misshapen bulbs and ruffled cuticles.

A 3-year-old Saudi girl presented to our clinic complaining, since early childhood, of slowly growing unruly scalp hair. The patient has black-colored hair that can be easily and painlessly pulled out with minor traction or combing. Her mother described that the patient always had short hair and never required a haircut. There is however no consanguinity between the parents and no similar family condition, including her 3 older brothers, who have easily combable diffuse hair that requires a regular haircut. Upon clinical examination, there were no signs of systemic associations with no abnormal nails, teeth, or skin findings. There were no complete alopecic areas localized on rubbing or traction zones, however, the hair pull test was positive, and the light microscopic examination of the plucked hairs revealed misshapen anagen bulbs and ruffled appearance of the cuticles (Figures 1 and 2). Based on this finding, a diagnosis of LAHS has been made.

## Conclusions

LAHS usually affects the hair of the scalp and rarely the body hair. It clinically manifests as fine, sparse, or unmanageably unruly hair texture that is poorly growing and rarely requires haircutting. Additionally, a 3 phenotypic presentation of LAHS was observed, including slowly growing sparse hair, diffuse or patchy unruly hair, and excessive shedding in normally appearing hair [1, 2].

LAHS has an autosomal dominant inheritance, and a mutation in *K6HF* and *K6IRS* genes encoding for keratin was found in some families. However, sporadic cases of LAHS were also described [1, 2].

The diagnosis of LAHS is based on the clinical and light microscopic examination of the hair that shows misshapen bulbs and ruffled cuticles. However, there is no associated abnormal laboratory finding in most patients [1, 2]. LAHS has an isolated presentation in most cases. Nevertheless, an association with other disorders is reported, including Noonan syndrome, uncombable hair syndrome, hypohidrotic ectodermal dysplasia, and nail-patella syndrome [1, 2]. LAHS has a good prognosis with spontaneous improvement during adolescence or adulthood, and topical minoxidil can be used as first-line therapy [2].

There are limited black-haired children reported with LAHS in Egypt, India, an African American girl, and a South-Asian boy [1, 2]. To our knowledge, this is the first case of LAHS of a child with black-colored hair from Saudi Arabia and the Gulf area.

LAHS in black-haired patients was seldomly described; however, similar cases could be underdiagnosed. Physicians probably rule this condition out since it is frequently connected to light-skin-colored children.

## References

[1] Dey V, Thawani M (2013). Loose anagen hair syndrome in blacked-haired Indian children. Pediatr Dermatol.

[2] Leerunyakul K, Suchonwanit P (2019). A Case of Loose Anagen Hair Syndrome in a Southeast Asian Boy. Case Rep Dermatol.

